# Development of Biodegradable Bioplastics with Sericin and Gelatin from Silk Cocoons and Fish Waste

**DOI:** 10.3390/toxics12070453

**Published:** 2024-06-24

**Authors:** Natesan Vijayakumar, Aathiyur Velumani Sanjay, Khalid A. Al-Ghanim, Marcello Nicoletti, Gurunathan Baskar, Ranvijay Kumar, Marimuthu Govindarajan

**Affiliations:** 1 Department of Biochemistry and Biotechnology, Faculty of Science, Annamalai University, Annamalainagar 608002, Tamil Nadu, India; nvkbiochem@yahoo.co.in (N.V.); avsanjay1997@gmail.com (A.V.S.); 2 Department of Zoology, College of Science, King Saud University, Riyadh 11451, Saudi Arabia; kghanim@ksu.edu.sa; 3 Department of Environmental Biology, In Unam Sapientiam, Sapienza University of Rome, 00185 Rome, Italy; marcello.nicoletti@uniroma1.it; 4 Department of Biotechnology, St. Joseph’s College of Engineering, Chennai 600119, Tamil Nadu, India; basg2004@gmail.com; 5 School of Engineering, Lebanese American University, Byblos 1102 2801, Lebanon; 6 University Centre for Research and Development, Department of Mechanical Engineering, Chandigarh University, Mohali 140413, Punjab, India; ranvijay.e9210@cumail.in; 7 Unit of Vector Control, Phytochemistry and Nanotechnology, Department of Zoology, Annamalai University, Annamalainagar 608002, Tamil Nadu, India; 8 Department of Zoology, Government College for Women (Autonomous), Kumbakonam 612001, Tamil Nadu, India

**Keywords:** sericin, gelatin, biomaterials, bioplastics, cellulose nanocrystals

## Abstract

The bioplastics sector promotes environmentally friendly means of cutting down on the usage of fossil fuels, plastic waste, and environmental pollution. Plastic contamination has detrimental effects on both ecological systems and the global food supply. The approach we present here to resolve this issue involves the integration of sericin and gelatin, obtained from cocoon and fish waste, respectively, with nano-reinforced cellulose crystals, to develop a biodegradable and compostable plastic material. The use of cocoon and fish wastes for the extraction of sericin and gelatin presents an environmentally beneficial approach since it contributes to waste reduction. The sericin level found in silk cocoon waste was determined to be 28.08%, and the gelatin amount in fish waste was measured to be 58.25%. The inclusion of sericin and gelatin in bioplastics was accompanied by the incorporation of glycerol, vinegar, starch, sodium hydroxide, and other coloring agents. Fourier transform infrared (FTIR) examination of bioplastics revealed the presence of functional groups that corresponded to the sericin and gelatin components. The tensile strength of the bioplastic material was measured to be 27.64 MPa/psi, while its thickness varied between 0.072 and 0.316 mm. The results of burial experiments indicated that the bioplastic material had a degradation rate of 85% after 14 days. The invention exhibits potential as a viable alternative for packaging, containment, and disposable plastic materials. The use of this sustainable approach is recommended for the extraction of sericin and gelatin from silk cocoons and fish waste, with the intention of using them as raw materials for bioplastic production.

## 1. Introduction

In recent decades, the utilization of traditional polymers has significantly increased [1]. Because of their adaptability to a wide range of uses, plastics derived from petroleum are becoming more integrated into human existence [2,3,4]. In this scenario, it is difficult to entirely eradicate the use of plastic from the human lifestyle, though the non-degradability of this petroleum-based plastic and its by-products presents a severe environmental concern [4] and it is difficult to effectively manage this solid waste [5]. Many synthetic plastics, such as polystyrene, polyethylene terephthalate, polyethylene, polyvinyl chloride, polypropylene, and polyurethane, are highly resistant to natural breakdown [6]. Consequentially, the current use of non-degradable plastics and their derivatives causes soil contamination [7]. Additionally, aquatic habitats are highly influenced [8].

Plastics are difficult for microbes to break down owing to their large molecular weight, hydrophobicity, and chemical bond strength [9]. To remedy this, conventional plastics require a biodegradable replacement. In that context, researchers have recently discovered the advantages of bioplastics, such as their biocompatibility, low toxicity, biodegradability, and renewability. Biodegradable plastics are designed to break down naturally over time, potentially reducing their environmental impact compared to traditional plastics. They can be made from renewable resources, like cornstarch, vegetable oils, or cellulose, and they have the potential to degrade into harmless by-products through biological processes. By using biodegradable alternatives for items like disposable cutlery, packaging, or bags, it is possible to reduce plastic pollution and alleviate some of the environmental issues associated with plastic waste [10].

Bioplastics are suitable for usage as plastic bags, disposable packaging films, buttons, and various items for food service. In the immediate future, it is anticipated that bioplastics will be developed as the next frontier. Several studies have identified renewable sources (from plants, such as polysaccharides and proteins, and from microbes, such as polylactic acid (PLA), polyhydroxyalkanoates (PHAs), poly-3-hydroxybutyrate (PHB), etc.) of raw materials that may be used in the most promising biodegradable plastics, with the same potential applications as synthetic or conventional plastics [11,12].

According to a previous statistical analysis, the global demand for bioplastics is projected to climb by 36% in the next five years [13]. The global yearly output of bioplastics is thought to have reached 2111 tons in 2020 and is anticipated to reach 2871 tons in 2025 [14]. However, bioplastics derived from microorganisms require extensive laboratory efforts to cultivate and maintain microbial cultures, as well as complex post-production processing. Moreover, the cultivation of crops for bioplastics, for example, can compete with food production and require the use of fertilizers and pesticides. Therefore, scientists are searching for other less costly methods for producing biopolymers, and agricultural, veterinary, and fishing wastes offer ideal raw materials [11].

The worldwide issue of the environmental effect of synthetic plastic was addressed by Surya et al. [15]. In order to provide a long-term solution to the plastic waste issue, their project investigated the production of bioplastics from fish scales and maize starch. This entailed the fabrication and in-depth examination of four distinct bioplastic films in order to assess their chemical and physical characteristics. Elsewhere, an informative analysis of fish scales as a special kind of natural biomaterial was given by Qin et al. [16]. The properties of fish scales, which consist of type I collagen and hydroxyapatite, were shown to closely resemble those of human hard tissues. As a result, they can be effectively utilized in a range of applications such as tissue engineering, wound healing, and sewage processing. The paper emphasized the capacity of fish scales to mitigate environmental strain and provide supplementary economic worth. The authors thoroughly examined the composition, microstructures, and many uses of these materials, with a particular focus on their potential to effectively tackle environmental and economic concerns.

Another study explored the feasibility of using high-protein waste materials derived from the fishing industry to produce environmentally degradable and biologically active films and coatings for food packaging, utilizing fish protein as the primary component. These cutting-edge solutions provided enhanced food quality, ecological sustainability, and economic worth. The use of additives improved their characteristics in favor of prolonging the lifespan of food and minimizing food wastage. The study offered a significant contribution to the advancement of sustainable packaging [17].

Gautam et al. [18] examined the use of fish waste and processing rejects to produce biodegradable films, using various additives. The film containing ascorbic acid (FGA) demonstrated a superior performance compared to the other films, exhibiting increased elasticity, tensile strength, and reduced water and oxygen permeability. FGA has remarkable mechanical and barrier qualities, making it an excellent choice for biodegradable food packaging. Pereira et al. [19] explored the influence of glycerol on films made from fish myofibrillar proteins. Decreased glycerol concentrations led to a decrease in the permeability of water vapor and an increase in the strength under tension. With an increase in glycerol concentration, the films exhibited enhanced elasticity but also showed elevated solubility. The films had distinct moisture sorption characteristics, and the GAB model accurately depicted the process within a defined range of relative humidity.

The uses of sericin, derived from silk cocoons, and gelatin, derived from waste fish products, are being investigated as a potential method for producing biodegradable and compostable polymers. In the garment business, the silk cocoon goes through a step called “degumming,” in which sericin is extracted and eliminated [20]. Approximately 30% of the total material in cocoons is sericin, which has a serine content elevation of about 32% [21]. In the field of sericulture, sericin has been ignored for a considerable time. On a worldwide scale, it is estimated that 400,000 metric tons of dried cocoons are generated, which will result in 50,000 metric tons of sericin. Most of this sericin is disposed of in effluent channels [22], leading to considerable water pollution and damage, in addition to a significant demand for biological and chemical oxygen in the environment.

The disposal of waste produced during the processing of fish might account for between 50 and 80% of the total raw material [23]. These types of wastes are a vital source of proteins for the production of meals that are high in protein content. The skin and bones of fish have a high concentration of collagen, which accounts for around 30% of the waste [24]. Hydrolysis of fish skin and bone collagen produces fish gelatin. This gelatin is similar to human-made gelatin [25,26]. Furthermore, the unique physicochemical properties of fish gelatin make it useful in the culinary and medicinal industries. 

In the past, a hydrogel was made from silk cocoons and fish excrements [27]. The production of bioplastics from cocoon waste combines sustainable practices and waste reduction, and fish waste, such as fish scales, skin, bones, and other by-products generated from the fishing and seafood processing industries, can be utilized to produce bioplastics with unique properties. Accordingly, the present study was focused on the fabrication of a bioplastic using organic wastes as renewable raw materials through a sustainable approach. The fabricated bioplastics were tested for their plasticity and decomposability when exposed to soil. This project adhered to the majority of the green chemistry principles, with the aim of producing no harmful by-products during manufacturing.

## 2. Materials and Methods

### 2.1. Sample Collection

The processed silk cocoons from textile industries and fish wastes from local fish slaughterhouses in and around the Namakkal district of Tamil Nadu, India were collected separately in clean glass containers. The samples were transported to the laboratory, rinsed individually three times with distilled water to eliminate contaminants, and then dried in a hot air oven at 65 °C for 24 h. The well-dried silk cocoons and fish waste samples were employed for sericin and gelatin extraction, respectively.

### 2.2. Extraction of Sericin from Cocoon

Sericin was extracted from the silk cocoons using a modified version of the method reported by Yang et al. [28]. Briefly, the well-dehydrated silk cocoon peduncles (10 g) were chopped into small pieces and soaked in 100 mL of extraction solution containing urea (8 M), sodium dodecyl-sulfate (SDS) (1%), and β-mercaptoethanol (2%) for 30 min at room temperature, and then heated for 5 min at 80 °C. The mixture was manually stirred during the heating process. The residual fiber was separated by filtration. The filtrate containing sericin was combined with 70% ethanol at a ratio of 1:3 and kept at −20 °C for one hour to yield sericin as a precipitate. All the chemicals were of analytical grade and purchased from Sisco Research Laboratories Pvt Ltd., Mumbai, India.

### 2.3. Qualitative and Quantitative Identification of Sericin

As a preliminary (qualitative) validation of sericin extracted from the silk cocoon, the weight-loss technique (sericin % = pre-extracted silk cocoon—post-extracted silk cocoon) and standard Biuret method were used. The extracted sericin was quantified using the standard Lowry’s technique [29].

### 2.4. Extraction of Gelatin from Fish Waste Cartilage

Fish waste cartilage was used to extract gelatin using a modified version of the method described by Shakila et al. [30]. Well-dehydrated fish wastes were treated (1:6 ratio g/mL) with 0.2% sodium hydroxide for 40 min to remove non-collagenous protein and rinsed with distilled water. The fish waste was treated (1:6 ratio g/mL) twice with 0.2% sulfuric acid to remove salts, rinsed with distilled water, and then treated with citric acid (1%) at the same ratio and washed with distilled water. Finally, the gelatin from this treated fish waste cartilage was extracted using distilled water (1:1 g/mL) and heated for 24 h at 45 °C. Then, the extract was filtered under vacuum using filter paper (Whatman No. 4). The filtered colloidal form gelatin was lyophilized and utilized for future investigations. The yield was calculated as (yield = [weight (g) of dried gelatin/weight (g) of the raw sample] × 100).

### 2.5. Fabrication of Bioplastics Using Sericin and Gelatin

Sericin taken from silk cocoons and gelatin derived from fish waste cartilage were combined with the optimal amounts of suitable additives. Using a mixer, 500 mg of sericin, 0.1 mL of gelatin, 0.7 mL of glycerol, 0.5 mL of vinegar, 0.5 g of starch, 7 mL of distilled water, 0.1 g of sodium hydroxide, and 0.05 g of color pigment were mixed entirely to produce a biocomposite powder. The well-blended biocomposite powder was placed in the extruder’s hopper (a long-heated chamber) (Thermo Scientific EuroLab 16 XL, Victoria, Australia). A moving screw progressively migrated the blends, and a rotating screw worked as a pump to press the molten plastic through the screw, releasing the bioplastic as a biocomposite film. With the assistance of a punching machine, the biocomposite film roll was converted into a bioplastic bag using the plastic-bag-making process.

### 2.6. Characterization of Fabricated Bioplastic Using FTIR and SEM Analysis

Fourier transform infrared spectroscopy (FTIR) is a powerful analytical technique used for the characterization of bioplastics. It provides valuable information about the chemical composition, structure, and functional groups present in biopolymer materials. FTIR can be used to determine the type of biopolymer used in the production of bioplastics. By comparing the FTIR spectrum of a bioplastic sample with reference spectra, the specific biopolymer fingerprint can be identified. This also provides insights into the molecular structure of bioplastics. Changes in the peak positions or intensities in the FTIR spectrum can indicate variations in the polymer structure [31]. The functional groups present in the fabricated bioplastic were examined using FTIR (IRAffinity-1S: Shimadzu, Kyoto, Japan) with the KBr pellet technique, and spectra were read between 4000 and 400 cm^−1^.

Scanning electron microscopy (SEM) is a valuable tool for characterizing bioplastics, allowing for a detailed analysis of their morphological features at a high magnification. SEM provides a detailed view of the surface structure of bioplastics, including several characteristics, such as roughness, porosity, and topographical variations. This information helps to assess the surface quality and determine the impact of processing conditions on the material’s final appearance and structure [31]. The surface structure and morphology of the fabricated bioplastic were evaluated using SEM analysis (VEGA3 TESCAN). An accelerated voltage of 25 kV was used to magnify the product’s surface by ×67.6K, and a crude component was infused afterwards [32].

### 2.7. Physical and Mechanical Properties of Fabricated Bioplastic

Investigating the surface quality, color, transparency, and presence of defects in bioplastics, as observed by visual inspection, is a basic but important method for assessing the quality of the fabricated bioplastic. Techniques reported by the ASTM standard were used to assess the thickness and tensile strength of the bioplastic formed by using sericin and gelatin. The thickness of a bioplastic provides valuable information about its physical characteristics. The thickness of the bioplastic was measured by using a caliper. Tensile testing was conducted using the ASTM D882 standard (Instron Micro tensile Tester, Model 5848) to determine the mechanical strengths of bioplastics, such as their tensile strength, elongation at break, and modulus of elasticity. All the tests were repeated two times and the mean values were reported [33,34].

### 2.8. Biodegradability of Fabricated Bioplastic

The microbial community of soil is capable of degrading organic compounds into nutrients, and considering biodegradability is essential when evaluating a biosynthesized product to replace conventional plastics. The biodegradability test determines the rate and extent to which microorganisms can break down bioplastics under specific environmental conditions. The biodegradability of the fabricated bioplastic was assessed using a soil burial test conducted at 30 °C under controlled moisture conditions [35].

## 3. Results and Discussion

In this study, sericin and gelatin were derived from discarded materials, that is, silk cocoons and fish by-products, to make bioplastics.

### 3.1. Extraction of Sericin and Gelatin

**Figure 1:**
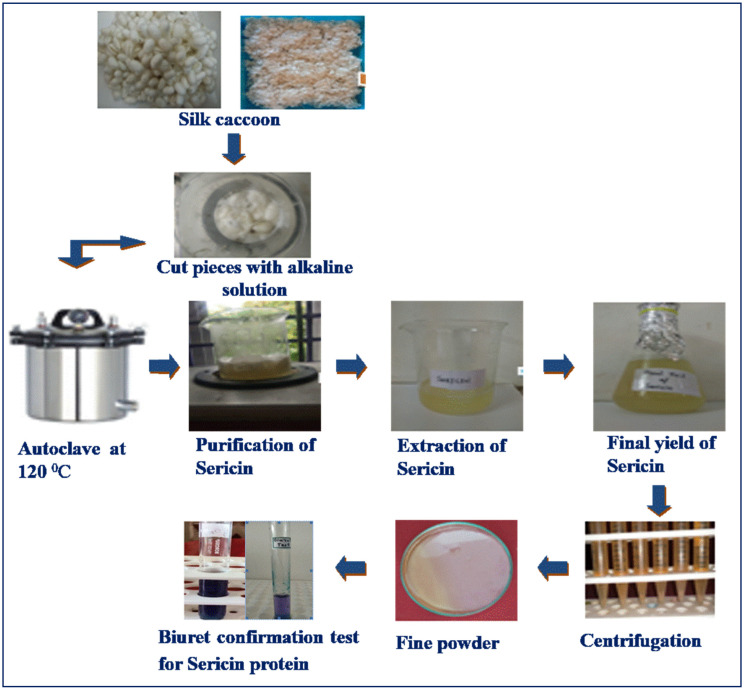
Process of extraction of sericin from silk cocoon for bioplastic production.

Through the weight loss estimate technique, we established that the silk cocoon obtained from textile industries produced 28.08% sericin. The findings of Lowry’s approach indicated a yield of around 29.56 mg/mL, which is a substantial amount when extracted from the silk cocoon. This finding is in accordance with the 25% extraction of silk sericin found by Wang et al., corresponding to about 28.2 mg/mL from cocoons of silk [36]. Meanwhile, a second report on sericin extraction from silk cocoons used an alkali-degraded process [37]; it was observed that sericin comprised 20–30% of the total silk cocoon mass, and this sericin was found to be a biopolymer containing roughly 32% serine. There are two types of silk sericins: α-sericin and β-sericin. They tend to be found in the cocoon’s outer and inner layers, respectively [20]. α-sericin has more N and O groups and fewer C and H groups [20,35]. Because sericin is easy to break down, it may be used to produce bioplastics (Figure 1).

**Figure 2:**
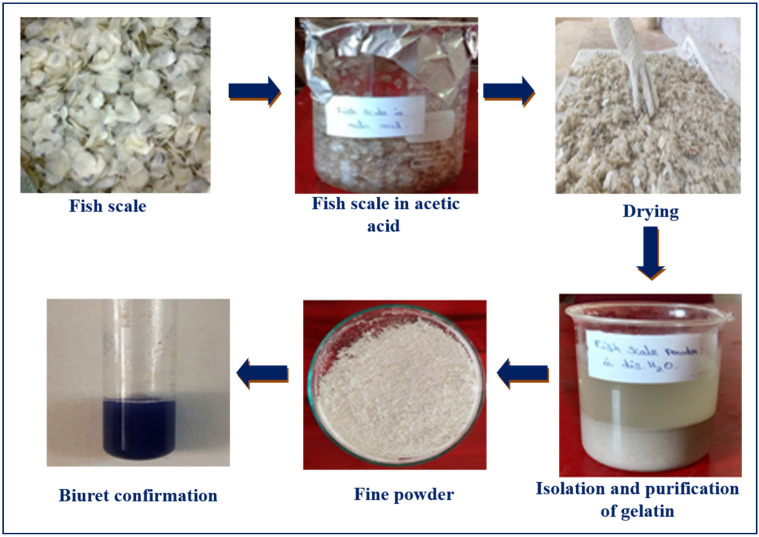
Process of gelatin extraction from fish waste cartilage for bioplastic production.

Meanwhile, gelatin was successfully sourced from fish waste, which included skins, bones, and scales (Figure 2). According to a previous scientific report, fish waste contains about 75% collagen compounds [10,38]. These collagen compounds are rich in fibrous structural proteins and can be used to make elastic products like bioplastics [39]. In particular, gelatin’s physical and chemical properties, such as its biocompatibility, stability, biodegradability, and mechanical strength, make it a promising material for producing bioplastics [40]. Beyond gelatin, further biodegradable components, including gluten, zein, and kafirin, derived from various biological sources, have been researched and determined to be appropriate for the production of bioplastics [41].

### 3.2. Fabrication of Bioplastics Using Sericin and Gelatin

**Figure 3:**
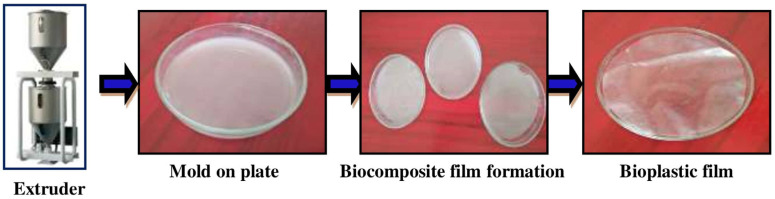
Lab-scale fabrication of bioplastic film through extrusion process.

The biocomposite film material was formed from suitable amounts of additives, like glycerol, vinegar, starch, sodium hydroxide, color pigment, sericin, and gelatin. Based on this appropriate combination, a fine biocomposite bioplastic film material was produced and heated in an extruder’s hopper. Then, the biocomposite material was shaped and made as thick as needed at room temperature (Figure 3). The long chain of polypeptides in gelatin gives it a fibrous structure that helps the film form. Mikhailov found that the biocomposite can create a triple helix structure when the temperature and other environmental conditions are correct [42]. Accordingly, gelatin has been used to produce packaging materials that are good for the environment and can be used for various edible products [43]. The starch added to this biocomposite material to produce a bioplastic can give gelatin and sericin strength and help with quick degradation through breakdown by soil microbes [44]. Note, however, that when producing a biocomposite, the additives change not only the physical, chemical, and mechanical properties of sericin and gelatin but also the biocomposite’s thickness, transparency, tensile strength, and ability to break down [45].

### 3.3. Characterization of Biocomposite Using FTIR Analysis

**Figure 4:**
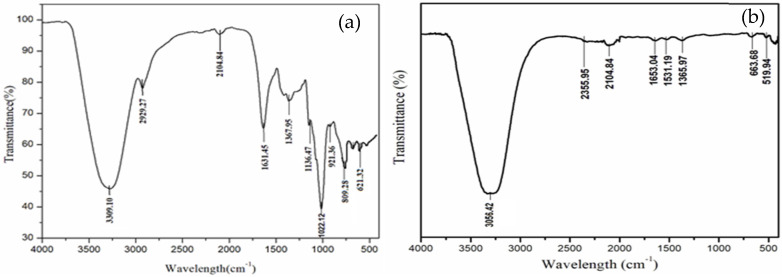
(**a**) FTIR analysis of biocomposite produced with gelatin; (**b**) FTIR analysis of biocomposite produced with sericin.

Fourier transform infrared (FTIR) analysis was used to examine the functional groups of the protein molecules in the final biocomposite product. The results showed that there were clear peaks for molecules related to sericin and additives (Figure 4a,b). The peak at 1699 cm^−1^ is assigned to the stretching vibration of C-O (amide I), and the peak at 1530 cm^−1^ to the stretching vibration of the secondary N-H bend (amide II). Also, the peaks at 1262 and 1245 cm^−1^, which Mostafa et al. [46] assigned to the C-N and N-H groups, strongly suggest that the biocomposite material used to make our bioplastic contained molecules that break down over time (Table 1). A previous FTIR analysis report of sericin from silk cocoons agreed with these results [47]. The peaks found between 3519 and 3708 cm^−1^ (like N-H and C-H stretching vibrations) were attributed to the functional groups of gelatin, starch, and other additives used in biocomposite formulations for making bioplastic [48].

### 3.4. Characterization of Fabricated Bioplastic Using SEM Analysis

**Figure 5:**
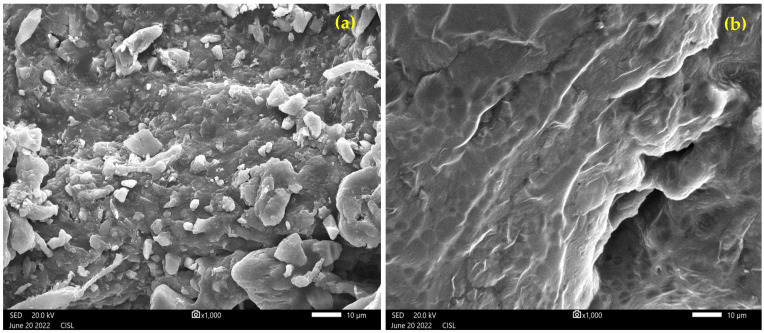
SEM analysis of surface morphology of bioplastic after (**a**) 7th day and (**b**) 21st day of burial test.

The degradation of sericin and gelatin in PBS, MES, and distilled H_2_O with 0.1% sodium azide solution was investigated on the 7th and 21st days at 37 °C. The results are shown in Figure 5a,b as a percentage of mass still present on the twenty-first day. Following 7 days, PBS had lost 50% of its weight. Degradation happened more rapidly in PBS than in MES or distilled H_2_O. Eventually, it was shown that PBS had a reduced rate of deterioration. PBS saw a 60% weight loss after 14 days, while MES and distilled H_2_O showed weight reductions of 43% and 83%, respectively. This suggests that the pH of the medium influences the rate of degradation. Thus, the SEM images in Figure 5 confirmed the changes in surface characteristics from rough and rigid on 7th day to softer and more compostable after 21 days of the test.

### 3.5. Physical and Mechanical Characteristics of Fabricated Bioplastic

Investigating the physicochemical features of bioplastic materials, such as thickness, transparency, tensile strength, and biodegradability, is crucial for evaluating a bioplastic’s quality and eco-friendliness [21].

#### 3.5.1. Biofilm Thickness and Transparency

**Figure 6:**
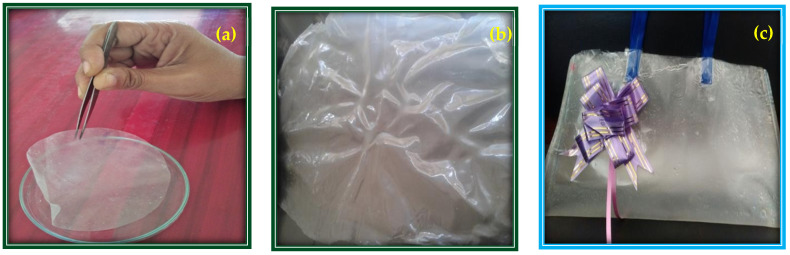
Characterization of sericin- and gelatin-based bioplastic: (**a**) biofilm thickness, (**b**) biofilm tensile strength, (**c**) final bioplastic bag.

Biofilm thickness is one of the physical factors that may influence the quality of bioplastics’ defining characteristics [6]. The consistency may be adapted to the desired products. Dense coatings improve strength but decrease the modulus of elasticity [49]. A test was conducted to determine the bioplastic thickness, including that with added sericin and gelatin. According to the findings of our bioplastic thickness tests for each treatment, the values of the four biofilms were determined to be 0.072 mm, 0.148 mm, 0.238 mm, and 0.316 mm, respectively (Figure 6a). When raising the concentration of an ingredient employed, the total dissolved solids incorporated in a biocomposite after dehydration will increase, resulting in a bioplastic with a greater thickness. The molding area may alter the thickness, biocomposite solution volume, and glycerol inclusion [50].

Transparency is an attribute that expresses how light passes through the material [51]. The key biocomposite elements, sericin and gelatin, contributed significantly to the biocomposite’s transparency. Figure 6a depicts the translucent appearance of the bioplastic film material. Clarity, which is inversely proportional to a film’s transparency rating [6], was somewhat lacking.

#### 3.5.2. Biofilm Tensile Strength

The bioplastic’s thickness, transparency, and tensile strength are the most important characteristics that determine its fabrication [31]. The tensile strength measurement indicates the force necessary to stretch a film to its maximum pull per unit area [52]. The tensile strength of our biocomposite bioplastic film containing sericin and gelatin was determined to be 21.03 MPa (Figure 6b). Figure 6b shows an image of the biofilm after testing its tensile strength. The bioplastic bag was composed of sericin and gelatin (Figure 6c) and could carry about 1.5 kg of products.

#### 3.5.3. Biodegradability of Fabricated Bioplastic

**Figure 7:**
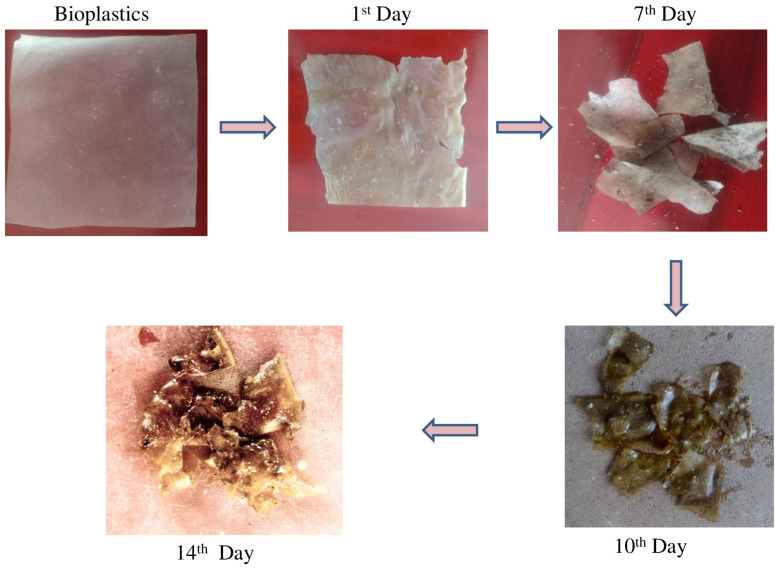
Biodegradation of bioplastic made with sericin and gelatin in 14-day burial test.

Four bioplastics were used to test the biodegradability of sericin- and gelatin-based film material (I–IV). This experiment revealed that the percentage of biodegradability after one day of burial ranged from 5.54 to 13.28%. Additionally, the percentage of decline on the seventh day ranged between 47.25 and 62.84% (Figure 7). The results are congruent with those reported by La Mantia et al. [35] and Degli Esposti et al. [53]. These researchers found that polyethylene glycol (PEG) improved the biodegradability of poly (DL-lactic acid) biopolymer and chitosan biofilms. Our results were more significant than those reported by Susilawati and coworkers [54]. In their study, on the first day, the biodegradation percentage of bioplastic made from tapioca flour combined with chitosan and fishbone-derived gelatin was between 3% and 4%. Meanwhile, the degradability percentage of the tested bioplastic films varied between 77.16 and 85.02% after 14 days of burial (Figure 7). Since gelatin is hydrophilic and sericin is hydrophobic, a higher gelatin content expedites bioplastics’ decomposition [23]. Biodegradable plastics’ hydrophilic properties attract microorganisms and make it easier for them to degrade and absorb nutrients [51]. In a short period, soil bacteria degraded the bioplastic composed of sericin and gelatin, effectively destroying the bioplastic.

## 4. Conclusions

Bioplastics offer several advantages over traditional fossil-fuel-based plastics, such as a reduced carbon footprint, decreased dependency on non-renewable resources, and improved waste management. They have the potential to significantly cut carbon emissions and our environmental impact, aiding in climate change mitigation. Ongoing research is enhancing their durability, strength, and heat resistance. Governments are increasingly promoting bioplastics through policies like bans and taxes on single-use plastics. For sustainability, it is essential to consider the entire lifecycle of bioplastics, including production, the waste management infrastructure, and consumer education. Investing in composting facilities and organic waste management is also crucial. Continued research, government support, and consumer awareness can help bioplastics play a key role in reducing plastic waste and fostering a sustainable future.

Compared to other technologies, sericin and gelatin offer significant advantages such as excellent biodegradability, biocompatibility, and cost-effectiveness due to their natural origins and the availability of raw materials. Sericin’s antioxidant properties and gelatin’s mechanical strength make them suitable for biomedical applications. However, sericin’s solubility and gelatin’s thermal instability pose challenges. Economically, using by-products from the silk and meat industries can reduce waste and production costs, meaning these biopolymers stand as sustainable and viable alternatives to synthetic polymers.

Silk cocoons proved to be an abundant source of sericin, comprising 28.08% of the material, and fish by-products comprising 58.25% gelatin also contributed significantly in our study. These extracted sericin and gelatin components were skillfully employed in the creation of bioplastics, further enriched by the incorporation of appropriate additives to enhance their properties. One of the most notable discoveries in our study was the remarkable biodegradability of the bioplastics we developed. Astonishingly, after just 14 days of testing, an impressive 89.02% of the bioplastic had already undergone significant degradation. This outstanding biodegradability highlights not only the potential of bioplastics crafted from sericin and gelatin but also the considerable environmental benefit they bring. These findings signify a promising alternative to conventional plastics, particularly at a time when plastic waste and its environmental impact are growing global concerns. The implications of this research extend beyond the laboratory, as it underscores the significance of bioplastics in promoting the much-needed transition from linear to circular economies within the plastics sector. By harnessing the potential of biodegradable and compostable bioplastics made from sericin and gelatin, we offer a sustainable solution that aligns with the broader goals of reducing plastic waste and advancing a more eco-conscious approach to material production. Our work contributes to the growing momentum in the field of sustainable materials and reinforces the critical role of bioplastics in driving this transformation.

## Figures and Tables

**Table 1:** FTIR peaks’ (values in cm^−1^) assignment of functional groups in bioplastic with sericin or gelatin.

Sericin	Gelatin	Peak Assignment
3056.42	3309.10	Amide-I
2355.95	2929.27	Amide-II
2104.84	2104.84	N-H bending of primary amines
1653.04	1631.45	C=O stretching of amide-I
1531.19	1367.95	N-H stretching of amide-III
1365.97	1136.47	C-N stretching of amines
663.68	809.28	C-O stretching of acid
519.94	621.32	C-O stretching of acid

## Data Availability

Data will be made available upon request.
